# Comparative effectiveness of manual therapy, pharmacological treatment, exercise therapy, and education for neck pain (COMPETE study): protocol of a systematic review with network meta-analysis

**DOI:** 10.1186/s13643-024-02737-4

**Published:** 2025-01-31

**Authors:** Ana Izabela Sobral de Oliveira-Souza, Jordana Barbosa-Silva, Douglas P. Gross, Bruno R. da Costa, Nikolaus Ballenberger, Tiago V. Pereira, Liz Dennett, Susan Armijo-Olivo

**Affiliations:** 1 University of Applied Sciences – Hochschule Osnabrück, Osnabrück, Germany; 2 Faculty of Rehabilitation Medicine, University of Alberta, Edmonton, Canada; 3 Clinical Trial Service Unit and Epidemiological Studies Unit (CTSU), Nuffield, Department of Population Health, University of Oxford, Oxford, UK; 4 Institute of Health Policy, Management and Evaluation, University of Toronto, Toronto, ON Canada; 5 Institute of Primary Health Care (BIHAM), University of Bern, Bern, Switzerland

**Keywords:** Neck pain, Manual Therapy, Exercise Therapy, Pharmacotherapy, Patient Education, Network Meta-Analysis

## Abstract

**Background and context of the study:**

Neck pain is a prevalent and globally burdensome problem. Clinical practice guidelines have recommended conservative treatments such as education, exercise therapy (ET), manual therapy (MT), and pharmacological therapy (i.e., medication) to manage all types of neck pain based on the chronicity of the disease (acute, subacute, and chronic pain). However, there is scarce evidence to determine which interventions constitute the most effective strategy for this condition.

**Research question:**

What are the best conservative treatment options (i.e., ET, MT, education, and/or medication) to relieve pain and disability-related outcomes in patients with neck pain?

**The overall purpose of the study:**

(1) To identify which type of conservative treatment (education, ET, MT, and/or medication) and their combinations have the greatest probability of being most effective for neck pain using a network meta-analysis (NMA) approach.

(2) To rank these conservative treatments in terms of safety (when possible) and effectiveness for managing neck pain.

**Methodology:**

Systematic review (SR) with NMA of randomized controlled trials (RCTs). Studies should include adults (aged > 18) with neck pain who received any of the interventions of interest (education, ET, MT, and medication). The main outcome will be pain intensity. Searches will be conducted in Ovid Medline All®, Embase, CINAHL (Cumulative Index to Nursing and Allied Health Literature), Scopus, and Cochrane Library Trials database. No language or publication date restrictions will be applied. The revised Cochrane Risk-of-Bias (RoB) tool for RCTs (RoB-2) will be used to evaluate RoB, and the certainty of evidence will be evaluated by Grading of Recommendations, Assessment, Development, and Evaluations (GRADE). NMAs will be conducted to rank interventions according to their effectiveness and safety (when possible), allowing a comprehensive analysis of all available evidence, with different nodes specified for all conservative interventions of interest, placebo, sham therapy, and non-intervention control.

**Major findings/summary of interpretations/conclusions:**

This NMA will help clinicians and the scientific community choose the most effective strategy or combinations of strategies for treating neck pain. The information gathered in this project will inform decision-making and guide personalized care of individual patients in the future.

**Supplementary Information:**

The online version contains supplementary material available at 10.1186/s13643-024-02737-4.

## Introduction

Neck pain is defined as a perceived pain anywhere in the posterior region of the cervical spine, from the superior nuchal line to the first thoracic spinous process [1]. Neck pain is one of the most prevalent and disabling health conditions in the world. It is considered the fourth largest contributor to global disability and within the top 20 most prevalent conditions worldwide [2]. The global prevalence of neck pain is 4.8% [3], and up to 70% of the general population will experience neck pain at least once, with a high percentage (between 50 and 80%) being recurrent [3]. The prevalence of neck/upper limb pain in European countries has been reported to be 44.6% (95%CI: 44.1, 45.1); this prevalence increases with age and is higher in women than in men (56% vs. 44%) and people with lower educational levels [4].

In addition to its high prevalence, neck pain is one of the top five most burdensome conditions in the world, with 33.6 million years lived with disability (YLDs) [3]. Neck pain and neck disability can adversely impact the quality of life, including family and social interaction, and it can affect the health system and the economy by interfering with daily activities and diminishing work productivity [5, 6]. The total cost of treatment for people with MSK pain, including neck pain, in Europe is estimated to be around €8.4 billion per year. Most (85%) of these costs result from lost work productivity (e.g., sick leave or presenteeism), and the other 15% are due to medical treatments [5]. Therefore, chronic musculoskeletal (MSK) pain, including neck pain, has been considered a major public health problem and a research priority in Europe and worldwide [7]. The frequency and intensity of neck pain increase sharply with age, contributing to an ever-growing burden of disease [8]. Thus, it is clear that neck pain is a burdensome problem globally, and effective management strategies are urgently needed.

Different types of treatment are available to manage people with neck pain [9–12]. Clinical practice guidelines have recommended conservative treatments such as exercise therapy (ET), manual therapy (MT), education, and pharmacological therapy to manage all types of neck pain based on the chronicity of the disease (acute, subacute, and chronic pain) [12]. In general, MT and ET are recommended for patients with acute and subacute neck pain, while for patients with chronic neck pain (CNP), a multimodal approach composed of MT, ET, medication, dry needling, or laser therapy has been recommended [4, 13]. In the last few years, education has also been considered a promising strategy to treat people with CNP, although little evidence is available [14, 15]. Thus, MT, localized or general ET, medication, and education have been described as good choices for treating patients with neck pain [4, 16–18]. However, there is not enough evidence or disagreements across guidelines regarding which interventions or combination of interventions constitutes the most effective strategy [4, 16, 17].

Although these therapies have been seen as good choices to treat neck pain, most available studies showing treatment benefits have compared these therapies with placebo, sham, or control interventions [19, 20]. These comparisons are the starting point to determine the effectiveness of an intervention strategy. However, in real-life situations, clinicians need to compare across therapies to guide decision-making. Unfortunately, there is not much scientific research comparing these therapies against each other (also called head-to-head comparisons) [19, 20]. Therefore, the decision-making processes are challenged due to the lack of direct evidence.

Due to the complexity of neck pain disorders and the decision-making process for neck pain conditions, traditional pairwise systematic reviews are of limited use. Network meta-analysis (NMA) has emerged in the last decades as a powerful statistical method for making head-to-head comparisons between interventions when there is insufficient direct evidence comparing therapies. This analysis technique can provide clinicians with the best combination of intervention components that present the best benefits for patients with a specific condition, representing and facilitating real-life decision-making [21].

Our research team has conducted several literature searches to find all NMAs for neck pain existing to date. This involved searching multiple databases (Cochrane Library, Epistemonikos, MEDLINE, and PROSPERO—the most important international registry for systematic reviews) for studies with relevant interventions. The searches revealed that three NMAs [22–24] have been published with completed results, five studies have been published as study protocols [10, 19–22], and nine protocols are registered in PROSPERO, with seven of these having an “in progress” status [10, 25–32]. The three published NMAs [22–24] on neck pain have exclusively addressed either ET or several therapies only for chronic neck pain. The NMA that focused on ET concluded that ET is better than no treatment for alleviating neck pain. However, there was limited evidence for some exercise therapies so that no meaningful conclusions could be drawn [22]. The second published NMA [23] focused on similar interventions that are treatments of interest in the present systematic review, but some relevant interventions (such as specific ET) were excluded from the analysis. In addition, only studies with chronic neck pain were included, and a limited number of medications were considered (only nonsteroidal anti-inflammatory drugs (NSAIDs). The third and most recent NMA [24] focused only on mind–body exercise interventions, including yoga, Pilates, Qigong, and Tai Chi. The results showed that isolated yoga was considered the best technique to improve neck mobility, and Pilates was the best exercise modality for improving mental health. However, the authors highlight that more high-quality evidence is needed to understand the comparative effectiveness of different mind–body exercise interventions for chronic neck pain [24].

Six different NMA protocols focused on neck pain were found [10, 22–25, 33]; however, they have different eligibility criteria compared to the present NMA protocol. Three protocols focus on only *one* of the four main interventions of interest in this review (ET, MT, education, and medication) [10, 33, 34]. Of the two protocols that included all the interventions of interest, one focused only on chronic neck pain, and the other did not specify whether they targeted acute/subacute or chronic pain. Moreover, one protocol restricted the eligibility criteria by targeting only middle-aged and older adults [35]. In addition, the approach to synthesis and analysis of the information of these protocols was superficially described, making it unclear how the information will be clearly synthesized to improve decision-making. In addition, the protocols did not include an analysis of components, that is, whether they will analyze the effect of treatment combinations and how authors will separate different intervention strategies into meaningful categories (nodes) [35].

Therefore, the scope of the NMAs planned by other research teams is reduced compared with the NMA planned in the present project. Our preliminary search revealed many additional relevant studies that were not included in previous NMAs. This lack of granularity in the literature does not contribute to the decision-making process. Rehabilitation professionals need to know, for example, whether motor control is better than aerobic exercise, or if strengthening exercise is better than MT, or when a combination of therapies would be optimal for treating neck pain in a specific group of patients. Also, physicians would benefit from knowing how the effectiveness of ET, MT, or education compares to medication. If ET or MT are found to be equally effective as medication, then it would be advisable to use them as a treatment strategy since they result in fewer adverse events than medication, which could help maximize adherence and effectiveness. Therefore, findings from this NMA may provide a more comprehensive view of the evidence and improve decision-making for several health professionals and inform real-life clinical decisions.

This review has the following objectives: (1) to identify which type of conservative treatment (e.g., exercise therapy (ET), manual therapy (MT), education, and pharmacological therapy) and/or their combinations has the greatest probability of being most effective for neck pain using a network meta-analysis approach, (2) to rank conservative treatments in terms of safety (when possible) and effectiveness for managing neck pain, and (3) to explore subgroup effects to identify people who are more likely to benefit from each treatment.

## Methods

A systematic review protocol was developed and registered in the PROSPERO database in April 2024 (CRD42024537623). It was prepared according to the Preferred Reporting Items for Systematic Reviews and Meta-Analyses Protocol (PRISMA-P) guidelines. To structure the contents of the actual systematic review and NMA, we have completed the PRISMA-P checklist (Appendix 1) and the PRISMA-NMA extension statement.

### Inclusion and exclusion criteria

The inclusion and exclusion criteria were developed based on the PICOS structure.

#### Population

We will include trials examining male and female adults (mean age > 18 years) with acute (less than 30 days), subacute (> 30 days and < 90 days), or chronic (non-specific) neck pain (> 90 days) of musculoskeletal origin**,** defined as pain perceived anywhere in the posterior region of the cervical spine, from the superior nuchal line to the first thoracic spinous process. Studies will be excluded if they involve patients with a mean age under 18 years of age or patients with neurological, rheumatic, vascular, metabolic diseases, cancer, previous surgery, or pain that is not clearly related to the MSK system. By excluding these conditions, we will ensure a more homogenous set of patients. If trials present a mixture of different populations, we will include the study as long as the information can be extracted for the adult population of interest.

#### Interventions

We will focus on potentially effective interventions to target neck pain, which have been recommended in clinical practice guidelines for different neck pain conditions [36]. In addition, these therapies are generally used as a first line of treatment to manage neck pain, and they are most likely used in clinical practice when treating patients with these conditions. Studies will be included if they investigated any of the following therapies: (1) ET (e.g., strengthening, aerobic exercise, motor control/stabilization, water exercises, among others), (2) manual therapy (e.g., mobilization techniques, manipulation techniques, massage, among others), (3) pharmacological therapy (non-steroidal anti-inflammatory drugs (NSAIDs), analgesics (e.g., paracetamol), opioids, among others), and (4) general education and/or pain neuroscience education (PNE) (i.e., educational sessions that describe the neurobiology and neurophysiology of pain by the nervous system) to manage neck pain. We will extract intervention details as suggested by the Template for Intervention Description and Replication (TIDieR) checklist to create consistent nodes to be used in NMA [37]. Furthermore, we will use similar classification frameworks as previous NMAs to classify the interventions of interest.

The process of creating the nodes and classifying the treatments will be iterative and will be performed independently by two reviewers during data extraction. Both reviewers will classify the intervention of interest during data extraction by following a pre-specified list created by the research group, containing the description and classification of different types of prescription of the main four conditions of interest (i.e., ET, MT, education, and medication) (Table 1). Based on the description provided by the authors in the primary studies, interventions will be classified, and the reviewers will generate nodes. Single and combined treatments will be included and grouped into nodes according to similarities (see Table 1 of classification/potential nodes description). Disagreements in nodes’ classification between reviewers will be settled, and the consensus between reviewers on nodes classification will be used for further refinement if needed. A committee within our team will be created to ensure nodes are consistent, accurate, and useful.

**Table 1:** Definitions of interventions based on the general base of treatment

**Node**	Definition and examples
**Exercise therapy (ET)**	
Strengthening/resistance	Exercise training designed to improve the strength, power, endurance, and size of skeletal muscles
Stretching	Exercise training including muscle lengthening using any of the following methods: passive, static, isometric, ballistic, or proprioceptive neuromuscular facilitation
Stabilization/motor control	Exercise training targeting specific trunk/neck muscles to improve control and coordination of neck and related structures
Proprioception	Exercises to increase the proprioceptive and kinaesthetic control
Pilates	Exercise training following traditional Pilates’s principles such as centring, concentration, control, precision, flow, and breathing
Yoga/Tai Chi/Qigong	Exercise training following traditional yoga/Tai Chi/Qigong principles with a physical component
Aerobic supervised training	Exercise’s training such as walking, cycling, and jogging in any land-based mode that is designed to improve the efficiency and capacity of the cardiorespiratory system guided by a health professional and conducted generally in a clinical setting
Water-based	Exercise training performed in deep or shallow water
Prescribed physical activity	General recommendation to perform exercises such as walking, cycling, and rowing, aimed at improving overall physical activity. These recommendations are not supervised and are carry out by the participants on their own
Balance	Exercises aimed at improving postural balance
Relaxation	Techniques that reduce stress and promote calm (i.e., deep breathing, muscle relaxation, and meditation)
Respiratory exercises	Exercises focus on improving the function of the lungs and respiratory muscles by controlling breathing patterns (i.e., diaphragmatic breathing, pursed-lip breathing, and controlled breath holding)
Postural exercises	Movements that strengthen the muscles involved in maintaining proper postural alignment (such as the core, back, and pelvic muscles), while also increasing flexibility and balance
Ergonomic	Ergonomic exercises are specific physical activities aimed at preventing and alleviating musculoskeletal complains and repetitive stress injuries by improving body mechanics and promoting proper alignment. They focus on enhancing flexibility, strength, and posture of muscles and joints, particularly those most affected by prolonged sitting, typing, or repetitive motions
Multimodal	Two or more of the specific types of exercise training mentioned above (not deemed multimodal if only part of warm up or cool down)
Active exercise	Voluntary contraction and movement of muscles to perform physical activities, typically against gravity or resistance, without external help
Assistive exercise	Partially active movements in which the individual engages their muscles but requires assistance—either from a therapist, a device, or equipment—to complete the movement
Supervised exercise	Structured physical activity programs conducted with the direct supervision of a health practitioner who monitors the participant’s form, intensity, and progress
Not supervised exercise	Exercise not supervised by a health practitioner
Other	Exercise training that does not meet any of the specific types of exercise training mentioned above
**Manual therapy (MT)**	
Manual therapy: spinal manipulation	High velocity thrust techniques at or near the end of the passive or physiologic range of motion
Manual therapy: spinal mobilization	Low-grade velocity movement techniques within the patient’s range of motion and control
Neural mobilization	Techniques designed to facilitate the movement of neural tissue within its surrounding anatomical structures, such as muscles and fascia
Massage	Soft tissue massage, acupressure
Trigger point therapy	Soft tissue technique including only techniques associated with trigger point deactivation
Myofascial release	Movements applied gently with a sustained pressure to specific areas of the fascia to release restrictions and improve the body’s movement patterns
Maitland mobilization	Treatment of joints and soft tissues through skilled manual therapy techniques, which includes rolling, rotating, sliding, and separation traction
Mobilization with movement (MWM)	Mobilization with movement (MWM) is a manual therapy technique that integrates passive joint mobilization by the therapist with active movement performed by the patient. This method aims to immediately reduce pain and restore normal movement by applying a sustained, pain-free accessory glide to the joint while the patient actively moves through the impaired range of motion
Sustained natural apophyseal glides (SNAGs)	The application of an MWM in the spine is referred to as a SNAG. This manual therapy technique applied to the spine involves the combination of a sustained passive accessory glide (or joint mobilization) applied in the plane of the facet joints by the physiotherapist to the spine (specific motion segment) with active movement from the patient. SNAGs can be applied centrally on the spinous process or laterally on the articular pillar
Natural apophyseal glides (NAGs)	Painless oscillatory mid- to end-range mobilization applied in the plane of the facet joints on the spinous process or articular pillar applied between C2 and C7
Reverse NAGs	Painless oscillatory mid- to end-range mobilization is applied in the plane of the facet joints on the spinous process or articular pillar. This technique can be applied between C6 and the upper thoracic spine
High-velocity low amplitude (HVLA) technique	Rapid use of force over a short duration, distance, and/or rotational area within the anatomical range of motion of a joint to engage the restrictive barrier in one or more planes of motion to elicit the release of restriction
Passive accessory intervertebral movements (PAIVMS)	Passive accessory intervertebral movement to produce movements in directions that cannot be produced actively in isolation
Multimodal MT	Two or more of the specific types of MT techniques mentioned above
**Patient education**	
Pain neuroscience education (PNE)	Educational sessions that describe the neurobiology and neurophysiology of pain by the nervous system
Education	Educational intervention, advice on importance of staying active, reassurance among others
Behavioral graded activity (BGA)	Behavioral treatment integrating the concept of operant conditioning with exercise therapy comprising booster sessions
**Pharmacological**	
NSAIDs	Ibuprofen, naproxen, sulindac, ketoprofen, tolmetin, etodolac, fenoprofen, diclofenac, flurbiprofen, piroxicam, ketorolac, Indomethacin, meloxicam, nabumetone, oxaprozin, mefenamic acid, diflunisal, fenoprofen
Opioids (strong)	Morphine, hydromorphone, oxycodone, fentanyl, methadone, buprenorphine, diamorphine, tapentadol
Opioids (weak)	Codeine, hydrocodone, tramadol, pentazocine, tilidine
Muscle relaxants: benzodiazepines	Diazepam, estazolam, quazepam, alprazolam, chlordiazepoxide, clorazepate, lorazepam, flurazepam, clonazepam, temazepam, midazolam
Muscle relaxants: skeletal	Flupirtine, orphenadrine, dantrolene, carisoprodol, tizanidine, incobotulinumtoxinA, cyclobenzaprine, metaxalone, baclofen, methocarbamol, chlorzoxazone
Antidepressants	Duloxetine, desvenlafaxine, levomilnacipran, fluoxetine, fluvoxamine, paroxetine, escitalopram, citalopram, sertraline, amitriptyline, amoxapine, desipramine, imipramine, doxepin, clomipramine, trimipramine, protriptyline, imipramine, nortriptyline, doxepin, nortriptyline
Paracetamol	
Topical agents (non-opioid)	Diclofenac, capsaicin, lidocaine
**Main comparators**	
Control	No active treatment, no prescribed physical exercise, no physical/manual therapy. The waiting list is a good example of a control intervention
Oral placebo	Any treatment that has no active properties and is applied via oral (e.g., sugar pills)
Topical placebo	Any treatment that has no active properties and is applied on the skin (e.g., creams)
Sham therapy	An inactive procedure designed to mimic the active procedure as closely as possible (e.g., sham acupuncture, sham manual therapy)
Usual care	Any treatment that the targeted patient population would be expected to receive as part of the normal practice Usual care intervention may include information or general advice to stay active (without specific exercise instructions) or keep doing the treatment delivered by clinicians without study goals or protocolized treatments

Studies will be excluded if they include any electrotherapeutic resource (e.g., Interferential current, TENS), ultrasound, surgical approaches, herbal medicine, homeopathy, acupuncture, or dry needling as their sole intervention.

#### Comparator(s)

Any eligible therapy for this SR could potentially be used as a comparator; however, the main comparators would be true control groups (which means that no treatment was applied or participants were enrolled into a waiting list group), oral placebo, topical placebo, sham therapy (e.g., sham manual therapy), and usual care.

#### Outcomes

**Fig. 1:**
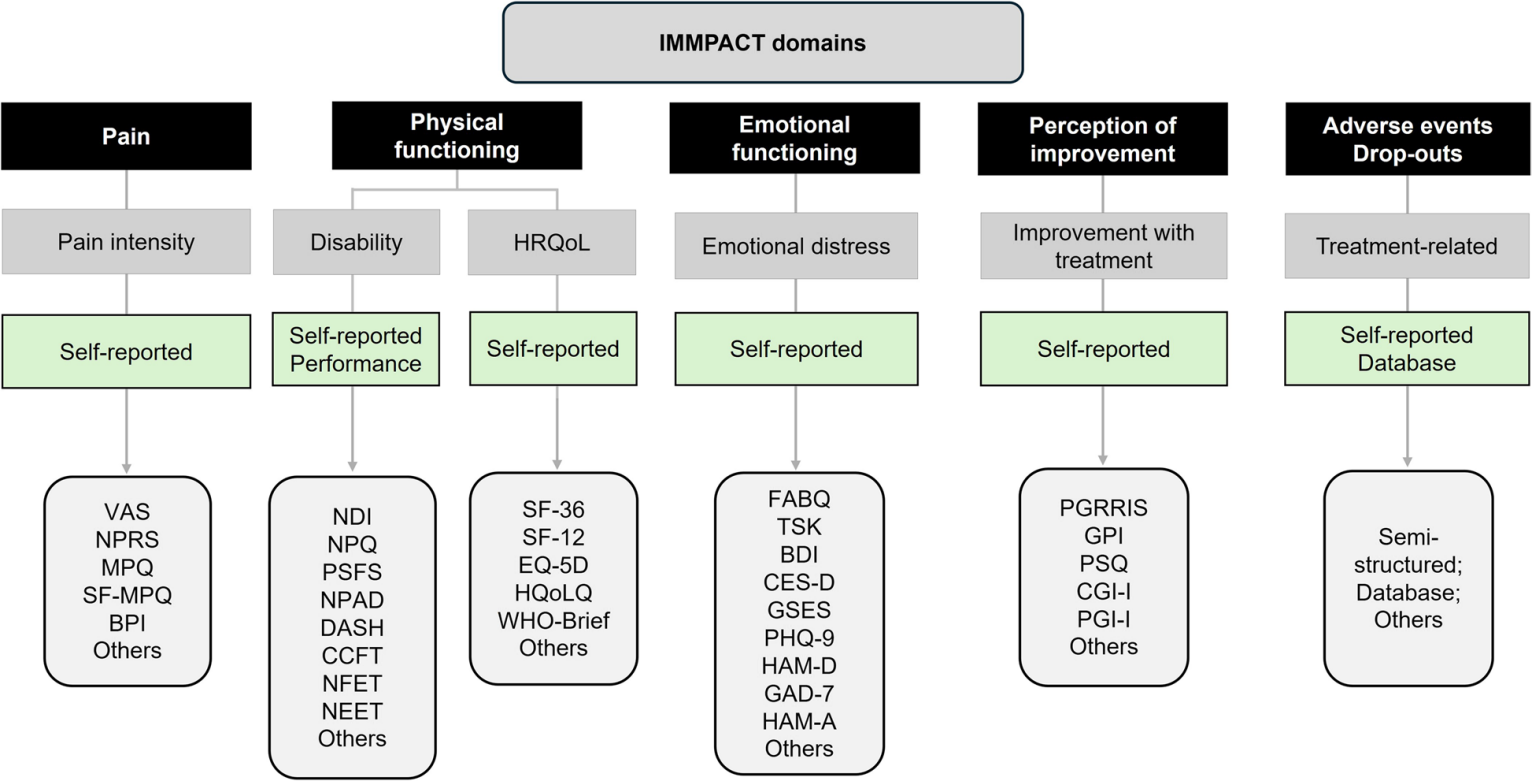
Overview of IMMPACT domains. NRS, Numerical Rating Scale; VAS, Visual Analog Scale; BPI, Brief Pain Inventory; NDI, Neck Disability Index; ODI, Oswestry Index; CCFT, Craniocervical Flexion Test; NFET, Neck Flexor Endurance Test; NEET, Neck Extensor Endurance Test; SF-36, 36-item Short Form Healthy Survey; BDI, Beck Depression Inventory; PHQ-9, Patient Health Questionnaire; HAM-D, Hamilton Depression Rating Scale; GAD-7, Generalized Anxiety Disorder; HAM-A, Hamilton Anxiety Scale; GPI, Global Perception of Improvement; PSQ, Patient Satisfaction Question; CGI-I, Clinical Global Impression – Improvement scale; PGI-I, Patient Global of Improvement

Based on the Initiative on Methods, Measurement, and Pain Assessment in Clinical Trials (IMMPACT) recommendations [38], the assessment of multiple outcome domains is required to determine the effectiveness of a treatment targeting pain adequately. The IMMPACT consensus recommends core outcome domains. These domains will be considered for this review. Studies will be required to include at least one of the outcomes of interest (the list of instruments that could be used to measure these outcomes—but not restricted to—are described in Fig. 1):

1. *Pain intensity*: This is the primary outcome of this review. Pain intensity could be measured in different ways (e.g., pain at the moment, pain in the last week, worst pain, general pain in the last month). Authors could also use different tools to measure pain intensity (as described in Fig. 1). When a trial reports that pain intensity was measured by two or more different scales/instruments/tools, results included in the network meta-analysis will be those reported using the scale/instrument/tool that has the highest hierarchy in our pre-specified hierarchy criteria. These hierarchy criteria were developed based on an international survey that identified the best outcome measures to be used in clinical practice for informing decision-making [32, 33]. An analogous hierarchy was developed for all IMMPACT domains of this review. For more details of this hierarchy, please see Fig. 1.
  The following domains described by the IMMPACT will be included as secondary outcomes:
2. *Physical functioning*. This domain includes assessment of diverse aspects of a participant´s life, such as the ability to carry out daily activities (e.g., walking, dressing, self-care) and specific measures of strength and endurance. It could be evaluated by physical tests (e.g., cranio-cervical flexion test) or by questionnaires (e.g., Neck Disability Index (NDI)). This domain also includes health-related quality of life (HRQoL) assessment, which refers to how a person feels and functions in their daily activities; specific questionnaires could measure this outcome (Fig. 1).
3. *Emotional functioning*. This domain includes assessing emotional distress, such as depression, anxiety, anger, and irritability. Many questionnaires, such as the Beck Depression Inventory, could be used to assess this outcome.
4. *Global perception of improvement*. This domain assesses the patient’s perception of improvement after treatment. Normally, patients must answer a questionnaire developed based on the Likert scale to classify their perception related to their symptoms (e.g., neck pain) after treatment.
5. *Adverse events/dropouts*. This domain includes any treatment-emergent adverse events and dropouts (any cause) that could arise during any therapy involved in this review. *Timing*: The following time points will be assessed: (1) end of treatment (main time point of interest), (2) short-term (2–6 weeks after treatment), (3) short-to-intermediate (7–12 weeks), (4) intermediate (≥ 12 weeks– ≤ 52 weeks), and (5) long-term follow-up (≥ 52 weeks). The results of the studies will be organized around these time points as much as possible, based on the number of studies and the timepoints we found in the included studies. Only effectiveness/efficacy outcomes will be assessed at multiple time points. For safety outcomes, we will focus on the final follow-up or the end of the trial. If this classification proves unhelpful, we will modify these time points to reflect the literature. *Designs*: The present systematic review will include only RCTs since they are the best designs to determine the effectiveness of interventions. Cross-over trials will also be included, but only the first period will be included to avoid carry-over effects. Reviews (narrative or systematic) will not be included but will be checked for relevant studies. Quasi-randomized trials will be excluded.

### Search strategy

A health sciences librarian with more than 15 years of experience in conducting systematic reviews will conduct searches in the following databases: OVID Medline All ®, Embase (Ovid Interface), CINAHL Plus with Full Text (Ebsco Interface), Scopus, Cochrane Library Trials database (Wiley Interface) from the date of inception. The search terms were developed using an iterative process; keywords identified by our team from the literature, as well as using algorithms from the “R packages” (litsearchr y bibliometrix), identified the best mapping words for our research question. Animal studies will be removed, and study design will be limited to RCTs. No date or language limits will be applied to the search. For any languages our multilingual team is not capable of reading, we will use professional translators to translate articles into English. The reference lists of included studies will be searched. In addition, Scopus or Web of Science will be used to track references and citations from included trials. An example of a search strategy conducted in Ovid Medline All ® (1946-March 21, 2024) is presented in Appendix 2.

### Study selection

The number of included studies will depend on the availability of published articles, but we anticipate around 300–400 studies to be included based on preliminary searches and screening performed by our team. Search results will be imported to Covidence (www.covidence.org), which will be used for the screening process. The PRISMA flow chart will be used to organize and keep track of the selection process. Two independent reviewers with expertise in conducting systematic reviews and research in MSK disorders (guided by the principal investigator and study team) will screen the titles, abstracts, and full texts of all the studies obtained from the searches, adhering to the inclusion and exclusion criteria developed and described above. If discrepancies occur between reviewers, a consensus meeting will be performed. In case of disagreement between reviewers, the principal investigator will make the final decision.

### Data extraction

Two reviewers will independently extract the data from primary studies using a standardized data-extraction (DE) form that will be developed specifically for this review, using the Ragic platform (ragic.com). The DE form will be piloted and revised as needed through regular discussions and comparisons throughout a pilot DE process. Data extractors will also receive training to keep the process consistent. The elements of each selected article will include but are not limited to *article information* (e.g., year of publication, country, language, funding, trial register), *study information* (e.g., main objective, study design, sample characteristics (population age, sex, diagnosis), data collection methods, total sample size, type of clinical trial, number of randomized groups), *treatment characteristics* (e.g., type of ET, type of MT, type of medication, type of education strategy, description of the treatment, compliance with the treatment, intervention fidelity analyses, parameters such as type, frequency, duration, and number of sessions applied), and *treatment classification* (see Table 1). We will also extract the *main and secondary outcomes*. These include a description of the outcome measurement tool (e.g., questionnaire, tests, measures) and how they were measured (e.g., cm, mm, points, score, among others). We will also extract the results, statistical tests, and the studies’ conclusions. In addition, we will extract *quantitative data* from the selected studies, such as mean (or median), standard deviation (or interquartile range), and 95% confidence intervals (CI) of all reported outcomes. If the outcomes are presented dichotomously, we will collect the number of events for each group (treatment and control groups) and risk ratios or odds ratios and their respective 95%CI. When the outcomes are continuous, effect sizes (ES) will be presented as mean differences (MD) or standardized mean differences (SMD). All quantitative data will be extracted at baseline, end of treatment programs, and follow-ups. To analyze continuous outcomes, we will use change scores from baseline as suggested previously [39]. When change scores are unavailable or cannot be approximated, post-treatment data will be used when possible. For effectiveness outcomes, we will give preference to results based on intention-to-treat (ITT) analyses over per-protocol (PP) or treated (AT) analyses. However, when ITT estimates are not reported, we will conduct analyses with available information and will note this in our reported results.

We will contact authors to obtain more detailed information at least three times when necessary (i.e., when reported data are incomplete or insufficient). After the third attempt, if data cannot be obtained, the study will not be included in the quantitative analysis. In case of disagreement between reviewers in the extracted data, the principal investigator will make the final decision. Consensus data will be used for all analyses.

### Risk of bias assessment

Two independent reviewers will assess the risk of bias using the Cochrane Revised Cochrane Risk-of-Bias tool for randomized trials (RoB-2) tool [40]. This tool contains the following five domains: randomization process, deviations from intended interventions, missing outcome data, measurement of the outcome, and selection of the reported result. For the overall assessment of the risk of bias for each study, studies will be rated as follows: *high risk of bias*, if the study was rated high in at least one domain; *some concerns*, if the study was rated with some concerns in at least one domain, and the other domains were low; and *low risk of bias*, if the study was rated as low risk in all individual domains. Previous studies have used similar decision rules when rating the overall RoB assessment of RCTs [10, 41]. Disagreements in risk assessment ratings will be resolved by consensus. Consensus ratings will be used for all analyses. RoB assessments will be included in the certainty of estimates of our NMA as suggested by the literature.

### Strategy for data synthesis

Data will be synthesized narratively and structured around neck pain chronicity (i.e., acute/subacute, chronic), type of treatment, and outcome measures (e.g., pain, physical, and emotional functioning). Evidence tables and figures will be used to present qualitative and quantitative data when appropriate. For the quantitative synthesis, respective effect sizes for the outcomes of interest will be computed. We anticipate that several tools will be used for our main outcome (pain intensity) and secondary outcomes, and therefore, we will use SMD as a metric.

Cohen criteria will be used to interpret the values of SMD found for our pooled estimates [42]. We will also analyze the minimal important difference (MID) for each outcome to interpret the summary treatment effects when possible following standard procedures and guidelines. For safety outcomes, treatment effects will be summarized with odds ratios.

We will conduct Bayesian NMAs to estimate the relative treatment effects based on direct and indirect evidence, and we will rank interventions according to their effectiveness and safety when possible. Summary treatment effect estimates will be obtained from the median and the corresponding 95% credibility intervals (CrIs) from the 2.5th and 97.5th percentile of the posterior distribution. For all Bayesian models, we will employ non-informative (vague) prior distributions for treatment effects. For the heterogeneity parameters, we will use empirical, minimally informative prior distributions [43]. We will conduct different analyses for different time points (as described in the “ Methods” section) and neck pain chronicity (i.e., acute/subacute, chronic); however, end-of-treatment data will be considered for the main analysis. This NMA will comprehensively analyze all available evidence, with different nodes specified (see Table 1). The relative effectiveness of all groups and treatments will be modeled against a reference treatment, mainly a control group (i.e., waiting list, no-treatment), placebo, or sham intervention. Rank plots, mean ranks, and Surface Under the Cumulative RAnking (SUCRA) will be used to rank interventions [44]. The outcomes and treatments that will be included in the NMA will be selected based on the characteristics of the available studies, and they will be presented graphically based on each outcome (e.g., pain, disability, other), in which nodes represent a class of intervention (as categorized in the nodes criteria, Table 1). We will check the transitivity assumption, which will be evaluated by comparing the distribution of potential effect modifiers across studies grouped by comparison [21]; preintervention pain and disability are considered as potential effect modifiers, which will be examined using boxplots or percentages to inspect potential effect modifiers of treatment effect visually. Incoherence will be assessed by using a stepwise approach [45]. We will first compare the model fit of coherence and incoherence models using the deviance information criterion (DIC) for an omnibus coherence assessment. If the incoherence model has a better DIC than the coherence model (e.g., an absolute difference greater than 5 in favor of the incoherence model), we will proceed with node-splitting to identify incoherent loops within the network. Model convergence will be performed with the Brooks-Gelman-Rubin statistic, trace plots, and autocorrelation plots [45]. Statistical heterogeneity will be assessed via the between-study variance and, if feasible, 95% prediction intervals. The analyses will be organized in several steps [46]: step 1, a network geometry will be developed to explore comparative relationships among interventions (e.g., MT, ET, pharmacotherapy, and education) organized by time point and pain chronicity; step 2, the coherence will be tested as explained above; step 3, all interventions will be ranked to identify superiority between them. Two approaches to determine the rank order of interventions are the SUCRA and the probability of being the best intervention. Due to the complexity of the data, multiple outcomes will be ranked; thus, an integrated ranking will be presented with pie charts [47] and rank-heat plot [48] when possible. Step 4, sensitivity analyses: a meta-regression will be conducted to assess whether heterogeneity can be explained by differences in studies in terms of clinical/methodological variables (at the trial level) such as overall RoB and Rob domains (when possible), sample size, and age groups, among others.

If feasible, we will assess small-study effects using funnel plots and statistical tests for funnel plot asymmetry [49, 50].

Due to the intricacy of the data, we expect complex interventions and a combination of treatments. Thus, if possible, we will conduct a component NMA in which the effect of each composite therapy will be expressed as the sum of the effects of its constituent components (additive model) or as an interaction of the constituent components (interaction model) as suggested in the literature [51, 52]. If subgroup effects are reported by the included trials (e.g., age, gender), we will try to compile similar subgroups across trials to explore subgroup effects in the networks when possible. We will use several software applications to perform the analyses (Stata, R, and MultiBUGS) [53] for this NMA. Step 5, the certainty of evidence produced by the synthesis for each outcome will be evaluated using GRADE [54]. A summary of the proposed analysis steps is provided in Table 2.

**Table 2:** Proposed analysis steps

Steps	Details
1. Data synthesis approach	Data will be categorized by: - Neck pain chronicity (acute/subacute, chronic) - Treatment nodes - Outcome (i.e., pain, physical, emotional functioning) Both qualitative and quantitative data will be synthesized using evidence tables and figures
2. Effect size calculation	Standardized mean difference (SMD) will be used for primary (pain intensity) and secondary outcomes, Cohen’s criteria will be used to interpret SMD values Minimal important difference (MID) will be considered to interpret mean differences (MD) obtained for each outcome, when possible
3. Time point and chronicity analysis	Analyses will be conducted separately for different time points and pain chronicity Main time point analysis will be “end-of-treatment data”
4. Adverse events analysis	Adverse events will be summarized using odds ratios (when possible)
5. Bayesian network meta-analysis (NMA)	Bayesian NMAs will estimate relative treatment effects based on direct and indirect evidence Interventions will be ranked by effectiveness using median values and 95% credibility intervals (CrIs) Non-informative prior distributions will be used for treatment effects; empirical priors will be used for heterogeneity parameters
6. Network geometry and comparison	To explore comparative relationships among interventions by time point and chronicity Treatments will be modeled against a reference (e.g., control group, placebo)
7. Ranking interventions	Rank plots, mean ranks, and SUCRA (Surface Under the Cumulative RAnking) will be used
8. Transitivity and incoherence testing	Transitivity will be checked by comparing potential effect modifiers across studies Incoherence will be assessed using the deviance information criterion (DIC) and node splitting for incoherent loops
9. Model convergence and heterogeneity	Convergence will be tested using the Brooks-Gelman-Rubin statistic, trace plots, and autocorrelation plots Heterogeneity will be assessed through between-study variance and 95% prediction intervals
10. Sensitivity analysis	Meta-regression will be used to explain heterogeneity in terms of clinical and methodological variables (e.g., RoB, age)
11. Subgroup analysis	Subgroup effects (e.g., age, gender) will be explored when possible

Due to the complexity of the analyses and literature, some of these analyses could change

## Discussion

This project will use a NMA to identify which type of conservative treatment (e.g., ET, MT, education, and pharmacological therapy) and/or their combinations have the greatest probability of being most effective for patients with acute, subacute, and chronic neck pain. To the authors’ knowledge, this is the first systematic review with an NMA that will combine all conservative treatment strategies recommended by clinical practice guidelines for neck pain.

Although several studies and systematic reviews have attempted to clarify the effectiveness of several therapies in isolation for neck pain (pair-wise meta-analyses), there is a scarcity of evidence about the comparative effectiveness of competing therapies for managing neck pain (also called head-to-head comparisons). NMAs have emerged in the last decades as a valid method of making head-to-head comparisons between interventions when there is insufficient direct evidence comparing these therapies. Previous published NMAs on neck pain only tell part of the story; none have successfully combined pharmacological and non-pharmacological treatments in a single analysis, which leaves an important gap in the body of evidence for decision-making, which our NMA will address.

The proposed systematic review with NMA has several methodological strengths. We will follow a rigorous methodological sequence, which includes the preparation of a protocol for the review, a systematic search of the databases, and the eligibility, data extraction, and quality of evidence of the studies that will be performed by two independents reviewers. We will follow NMA PRISMA’s recommendations, including analyzing the risk of bias in the included trials with the Cochrane RoB-2 tool, the most recognized tool for analyzing the risk of bias in clinical trial studies. Moreover, one of the strengths of our NMA protocol is that the process of combining the treatments into nodes will be based on a priori definitions of interventions previously published. The process of creating the nodes and classifying the treatments will be iterative and will be performed independently by two reviewers. Single and combined treatments will be included and grouped in nodes according to similarities (see Table 1). This classification will contribute to a more transparent selection and synthesis process. This is an important aspect since it has been described that less than 10% of NMAs published reports on how the selection process of the treatment nodes happened during the review [55, 56]. Thus, we will ensure that nodes are described before data collection. In addition, our project will rate the confidence of the evidence contributing to the estimation of interventions included in the network through the GRADE approach to facilitate the interpretation and uptake of findings [54].

However, there are some limitations to this protocol. Since we anticipate that a large number of trials will be included, this NMA will focus only on non-specific neck pain. No information will be provided for other types of neck pain. Also, a high heterogeneity between studies is expected due to the differences between treatment protocols, and most trials will likely have issues with methodological bias (based on our experience with this literature). Therefore, we anticipate conducting sensitivity analyses considering these issues when possible (e.g., low overall risk of bias vs. those with high or some concerns; large vs. non-large trials).

To conclude, findings from this systematic review with NMA will contribute substantially to the treatment decision-making process for one of the largest contributors to global disability, neck pain. We will optimize neck pain treatment by identifying the most effective conservative approaches, or combination, among the most used conservative treatment strategies for neck pain. This may result in a new direction for neck pain treatment since it brings a focus to interventions that would benefit the patient and can be successfully implemented in clinical practice. Moreover, our detailed statistical analysis will also allow clinicians and therapists to identify which group of patients might benefit from one type of intervention or combination of treatments. This knowledge will optimize the treatment of patients with neck pain. Moreover, results from this review may be used to generate benefit-harm analyses as conducted in different medical fields (i.e., cardiovascular). This will allow calculation of the probability that a patient will experience more benefit than harm from prescription of one or a combination of conservative approaches for neck pain. This knowledge will support individualized medicine, which is the future of health care. Therefore, this systematic review and NMA will fill an important gap in the literature and inform real-life clinical decision-making.

## Supplementary Information

Supplementary Material 1. Appendix 1: PRISMA 2020 Checklist.

Supplementary Material 2. Appendix 2: Search strategy.

## Data Availability

All data used by this review is available upon request.
