# Supplementary material for: Comparative effectiveness of manual therapy, pharmacological treatment, exercise therapy, and education for neck pain (COMPETE study): protocol of a systematic review with network meta-analysis
Source: Syst Rev. 2025 Jan 31;14:30. doi: 10.1186/s13643-024-02737-4 (PMC11786388; doi:10.1186/s13643-024-02737-4)
Supplement: Supplementary file 2 — Supplementary Material 2. Appendix 2: Search strategy. [file 13643_2024_2737_MOESM2_ESM.pdf]

SEARCH STRATEGY

Ovid MEDLINE(R) ALL <1946 to March 21, 2024>
Search date: March 22, 2024
Search saved as: COMPETE Medline 2024

https://login.ezproxy.library.ualberta.ca/login?url=http://ovidsp.ovid.com/ovidweb.cgi?T=JS&NEWS=N&PAGE=main&SHAREDSEARCHID=5dw8CTMFoYmbeyLbWUnWKbJembwLA3FB6ulF86mQHjyZgHUVHefb0r2eIYMORptV7

1 exp neck pain/ or Neck Injuries/ 14267
2 ((pain or ache or injur* or "disk degeneration" or "disk displacement" or "disk hernia" or "disk disease" or "disc degeneration" or "disc displacement" or "disc hernia" or "disc disease") adj10 ((neck not ("femoral neck" or "bladder neck" or "humeral neck" or "fibula neck")) or cervical or cervicothoracic or cervicogenic)).mp.49536
3 (("chronic pain" or "acute pain" or "subacute pain" or "intractable pain" or "persistent pain" or "musculoskeletal pain" or "noncancer pain" or "non-cancer pain" or "non-malignant pain" or "nonmalignant pain" or "nonneoplastic pain" or "non-neoplastic pain" or "myofascial pain" or "myofascial trigger point*") adj12 ((neck not ("femoral neck" or "bladder neck" or "humeral neck" or "fibula neck")) or cervical or cervicothoracic or cervicogenic)).mp. 1953
4 (pain/ or acute pain/ or chronic pain/ or musculoskeletal pain/ or myalgia/ or pain, intractable/ or pain, referred/ or Intervertebral Disc Degeneration/ or Intervertebral Disc Displacement/) and neck/ 1056
5 (neckache or (neck adj4 complaint*) or cervicodynia or cervicalgia or ((neck or cervical or cervicothoracic or cervicogenic) and (whiplash or radiculopathy or "zygapophyseal joint syndrome" or "facet joint syndrome"))).mp. 7803
6 ("neck disability index" or "Copenhagen Neck functional disability scale").mp. 3250
7 or/1-6 53726
8 (myelopath* or osteoporo* or RA or arthritis or SCI or spinal cord* or ((spine or spinal) adj2 fractur*) or parapleg* or quadripleg* or lupus or (cancer* not non-cancer) or (malignan* not non-malignan*) or oncolog* or neoplasm* or tumour* or tumor* or burn or burns or abuse* or donor or copd or chronic obstructive pulmonary disease or stroke).ti. 2698633
9 exp *Spinal Cord Injuries/ or exp *Osteoporosis/ or exp *Neoplasms/ or *arthritis, experimental/ or *arthritis, infectious/ or *arthritis, juvenile/ or *arthritis, psoriatic/ or exp *arthritis, rheumatoid/ or exp *rheumatic fever/ or exp *Lupus Erythematosus, Discoid/ or exp *Lupus Vasculitis, Central Nervous System/ or exp *Lupus Erythematosus, Cutaneous/ or exp *Lupus Nephritis/ or exp *Lupus Erythematosus, Systemic/ 3810946
10 7 not (8 or 9) 43040
11 exp Musculoskeletal Manipulations/ or Osteopathic Medicine/ or Physical Therapy Modalities/ or "physical and rehabilitation medicine"/ or rehabilitation/ 82257
12 (((Musculoskeletal or manual or postur* or myofunctional or manipulat*) adj8 therap*) or (neck adj8 adjust*)).mp. 23159
13 (manipulation* or mobilization* or mobilisation* or chiropract* or osteopathic or physiotherap* or physical therap* or pain-rehab* or active-rehab* or rehab*-program* or ((multimodal or interdisciplinary or multidisciplinary or group) adj3 rehab*) or massag* or masseuse* or passive jaw motion device or continuous passive motion or mulligan or Maitland* or Sustained Natural Apophyseal Glides or sustained neutral apophyseal glides or SNAGs or soft tissue technique* or trigger point therap* or diacutaneous fibrolysis or

kneating or rolfing or ischemic compression or crochetage or mckenzie* method).mp. 358532
14 11 or 12 or 13 387584
15 Patient Education as Topic/ or educat*.ti. 267240
16 ((educat* or explain* or video* or explanation or brochure* or pamphlet* or (website adj16 inform*) or app or mobile application or intervention*) and (pain adj5 (science or neurophysiolog* or neuroscien*))).mp. 736
17 ((educat* or explain* or video* or explanation or brochure* or pamphlet* or website or app or mobile application or intervention*) adj5 (neurophysiolog* or neuroscien*)).mp. and (pain.ti. or pain.hw.) 385
18 (pain education and (pain science or science of pain or pain biology or biology of pain or neurophysiolog* or neuroscien*)).mp. 99
19 (patient education or neck school* or alexander technique or pain neuroscience education).mp. 110377
20 15 or 16 or 17 or 18 or 19 287373
21 exercise/ or muscle stretching exercises/ or exp physical conditioning, human/ or preoperative exercise/ or running/ or jogging/ or swimming/ or walking/ or weight lifting/ or stair climbing/ or physical endurance/ or physical exertion/ or physical fitness/ or cardiorespiratory fitness/ or Dance Therapy/ or tai ji/ or yoga/ or exp exercise therapy/ or exercise movement techniques/ or drainage, postural/ or myofunctional therapy/ 357163
22 (exercis* or physical* activ* or physical* exert* or physical endurance or fitness or aerobic* or workout* or kinesiotherap* or stretches or stretching or core stability).mp. 790839
23 ((conditioning or strengthening) adj6 (program* or protocol* or regime* or strateg* or therap* or intervention* or progressive or functional or post-operative or postoperative or home or home-based or outpatient)).mp. 20905
24 ((train* adj6 (postur* or resist* or strength* or weight or stability or circuit* or interval* or endurance)) or (musc* adj6 (strengthen* or train* or contraction*)) or resistance activit* or progressive resist* or gravity resistive or stabilization exercis* or stabilisation exercis* or isotonic or isometric or ((eccentric or concentric) adj2 (contraction* or exercise*))).mp. 238949
25 (activat* adj4 muscle).mp. 20761
26 ((treadmill* not treadmill test) or (cardio* adj3 (class* or exercis* or train* or machine*)) or ergometer* or ergometre*).mp. 66386
27 (walking or ((walk or walks) adj3 (fast or brisk* or quickly or regular)) or swim* or running or jogging or cycling or bicycl* or bike or biking or spinning or spin class* or spin bike* or kickbox* or boxing or x-country ski* or cross-country-ski* or ((stair* adj3 climb*) not stair climb test)).mp. 379934
28 (rowing or skating or hiking or danc* or gymnastic* or calisthenic* or zumba or yoga or pilates or tai chi or tai ji).mp. 29444
29 (pedomet* or acceleromet* or Step-count* or count*-step* or Fitness-tracker*).mp. 30637
30 or/21-29 1305483
31 analgesics, opioid/ or alfentanil/ or alphaprodine/ or buprenorphine/ or buprenorphine, naloxone drug combination/ or butorphanol/ or codeine/ or dextromoramide/ or dextropropoxyphene/ or dihydromorphine/ or diphenoxylate/ or "enkephalin, ala(2)-mephe(4)-gly(5)-"/ or "enkephalin, d-penicillamine (2,5)-"/ or ethylketocyclazocine/ or ethylmorphine/ or etorphine/ or fentanyl/ or heroin/ or hydrocodone/ or hydromorphone/ or levorphanol/ or meperidine/ or meptazinol/ or methadone/ or methadyl acetate/ or morphine/ or nalbuphine/

or opiate alkaloids/ or opium/ or oxycodone/ or oxymorphone/ or pentazocine/ or phenazocine/ or phenoperidine/ or pirinitramide/ or promedol/ or remifentanil/ or sufentanil/ or tapentadol/ or tilidine/ or tramadol/ 139605
32 Baclofen/ 6037
33 Diclofenac/ 8959
34 anti-inflammatory agents, non-steroidal/ or "4,5-dihydro-1-(3-(trifluoromethyl)phenyl)-1h-pyrazol-3-amine"/ or adapalene/ or adapalene, benzoyl peroxide drug combination/ or ampyrone/ or antipyrine/ or apazone/ or aspirin/ or bufexamac/ or celecoxib/ or clonixin/ or curcumin/ or diclofenac/ or diflunisal/ or dipyrone/ or epirizole/ or etanercept/ or etodolac/ or etoricoxib/ or fenoprofen/ or feprazone/ or flurbiprofen/ or ibuprofen/ or indomethacin/ or ketoprofen/ or ketorolac/ or ketorolac tromethamine/ or meclofenamic acid/ or mefenamic acid/ or meloxicam/ or mesalamine/ or nabumetone/ or naproxen/ or niflumic acid/ or olopatadine hydrochloride/ or oxaprozin/ or oxyphenbutazone/ or phenylbutazone/ or piroxicam/ or salicylates/ or sodium salicylate/ or sulfasalazine/ or sulindac/ or suprofen/ or tolmetin/ or cyclooxygenase inhibitors/ 214250
35 (Antidepressants or SNRI? or (Serotonin adj3 reuptake inhibitor?) or Duloxetine or desvenlafaxine or levomilnacipran or venlafaxine or milnacipranor or SSRI? or Fluoxetine or fluvoxamine or paroxetine or escitalopram or citalopram or sertraline or vilazodone or tricyclic Amitriptyline or amoxapine or desipramine or imipramine or doxepin or clomipramine or trimipramine or protriptyline or imipramine or nortriptyline or doxepin or nortriptyline or NSAID? or non?steroidal anti?inflammat* or acetylsalicyl* or aspirin or Ibuprofen or naproxen or sulindac or ketoprofen or tolmetin or etodolac or fenoprofen or diclofenac or flurbiprofen or piroxicam or ketorolac or Indomethacin or meloxicam or nabumetone or oxaprozin or mefenamic acid or diflunisal or fenoprofen or opioid? or morphine or hydromorphone or oxycodone or fentanyl or methadone or buprenorphine or diamorphine or tapentadol or codeine or hydrocodone or tramadol or pentazocine or tilidineor or muscle relaxants or benzodiazepines or diazepam or estazolam or quazepam or alprazolam or chlordiazepoxide or clorazepate or lorazepam or flurazepam or clonazepam or temazepam or midazolam or skeletal flupirtin or orphenadrine or dantrolene or carisoprodol or tizanidine or incobotulinumtoxinA or cyclobenzaprine or metaxalone or baclofen or methocarbamol or chlorzoxazone or paracetamol or acetaminophen or tylenol or topical agent* or diclofenac or capsaicin or lidocaine).mp. 648893
36 or/31-35 745891
37 14 or 20 or 30 or 36 2607391
38 exp Clinical trial/ or (randomi* or randomly or (random adj4 (allocat* or distribut* or assign*)) or placebo or trial or groups or subgroups or (phase adj1 ("3" or "2" or "1" or III or II or I))).tw,kf. 4305530
39 ((case-control* or (cross-sectional not cross-sectional-area) or cohort or qualitative or (observational adj3 study) or case-series or case-report or case-study or delphi-study or bibliometric-analys* or questionnaire or survey or (tool and validat*)) not (rct or trial*)).ti. 1042790
40 38 not 39 4156158
41 10 and 37 and 40 3415
42 limit 41 to animals 37
43 limit 42 to humans 13
44 41 not (42 not 43) 3391
45 limit 44 to "review articles" 367

46	limit 45 to (clinical trial, phase i or clinical trial, phase ii or clinical trial, phase iii or clinical trial, phase iv or clinical trial protocol or clinical trial or meta analysis or "systematic review")	203
47	45 and (trial or random* or rct or rcts or systematic review or scoping review or meta-anal* or metaanal*).ti,bt.	184
48	44 not (45 not (46 or 47))	3287
